# Contextually-Based Social Attention Diverges across Covert and Overt Measures

**DOI:** 10.3390/vision3020029

**Published:** 2019-06-10

**Authors:** Effie J. Pereira, Elina Birmingham, Jelena Ristic

**Affiliations:** 1 Department of Psychology, McGill University, 1205 Dr. Penfield Avenue, Montreal, QC H3A 1B1, Canada; 2 Faculty of Education, Simon Fraser University, 8888 University Drive, Burnaby, BC V5A 1S6, Canada

**Keywords:** social attention, attentional biasing, faces, context

## Abstract

Humans spontaneously attend to social cues like faces and eyes. However, recent data show that this behavior is significantly weakened when visual content, such as luminance and configuration of internal features, as well as visual context, such as background and facial expression, are controlled. Here, we investigated attentional biasing elicited in response to information presented within appropriate background contexts. Using a dot-probe task, participants were presented with a face–house cue pair, with a person sitting in a room and a house positioned within a picture hanging on a wall. A response target occurred at the previous location of the eyes, mouth, top of the house, or bottom of the house. Experiment 1 measured covert attention by assessing manual responses while participants maintained central fixation. Experiment 2 measured overt attention by assessing eye movements using an eye tracker. The data from both experiments indicated no evidence of spontaneous attentional biasing towards faces or facial features in manual responses; however, an infrequent, though reliable, overt bias towards the eyes of faces emerged. Together, these findings suggest that contextually-based social information does not determine spontaneous social attentional biasing in manual measures, although it may act to facilitate oculomotor behavior.

## 1. Introduction

Faces convey a great deal of information. From an evolutionary perspective, researchers have theorized that the hierarchical system of social groups in both human and non-human primates primarily relied on visual information in faces to convey social signals to others [1,2]. As such, systems that processed these signals quickly and efficiently enhanced the ability to accurately predict other’s behavior and dispositions [3,4]. This prioritization of social information is evident developmentally, with a preference for faces and eyes early in life [5,6,7,8,9], as well as neurologically, with a distributed network of specialized brain structures within the temporal and occipital lobe (e.g., fusiform face area, superior temporal sulcus, occipital face area) that are specifically tuned for processing faces, gaze, and other socio-biological signals [10,11,12,13,14,15,16,17,18]. These findings suggest that information conveyed by faces and facial features like eyes represent a key component of the complex social communication system [19,20,21,22].

As such, it is intuitive to expect that faces and facial features would preferentially capture and spontaneously shift attention, a process often called social attentional biasing [12,23]. Consistent with this idea, research has demonstrated quick and spontaneous attentional biasing towards faces and eyes in both covert (attentional shifts independent of eye movements) and overt (attentional shifts accompanied by eye movements) measures. Covertly, attentional biasing is typically indexed by manual performance (i.e., response time and/or accuracy) that is elicited in response to targets that follow social versus non-social cues. Bindemann and colleagues [24] were among the first to show that attention is preferentially drawn to faces by presenting participants with side-by-side images of a face and a non-social object (e.g., a faucet) followed by targets that appeared equally often at either location. Even though participants had no incentive to shift their attention to either cue, faster responses were found for targets that occurred at the previous location of the face, suggesting that task-irrelevant faces spontaneously biased attention. Subsequently, similar effects have been reported using a wide range of popular behavioral paradigms (i.e., go/no-go tasks [25], rapid serial visual presentation tasks [26], visual search [27], change detection, and inattentional blindness paradigms [28,29]).

A spontaneous attentional bias for faces is also found when attention is indexed by the latency and/or pattern of eye movements occurring in response to social relative to non-social stimuli. Yarbus’ seminal work [30] provided one of the first demonstrations of an oculomotor bias for faces. He recorded participants’ eye movements while they freely viewed photographs of social scenes and found that they preferentially looked at faces and eyes. This general result has since been replicated by numerous studies, which collectively show that faces and facial features bias eye movements within the first two fixations [31,32,33,34] and elicit earlier saccades compared to other stimuli [35,36]. Similar overt social biasing has also been found in tasks that use controlled laboratory paradigms [37], those that manipulate static and dynamic representations of social behavior [38,39,40,41], as well as during tasks that measure social attention during dynamic real-life social interactions [42,43,44]. Thus, similar to covert attention, overt attention also appears to be spontaneously biased towards faces and eyes.

However, despite the abundance of evidence of an attentional bias towards faces, recent work has revealed that this effect may not be as robust as once thought. Pereira, Birmingham, and Ristic [45] noted that previous studies reporting an attentional bias for faces lacked rigorous control over stimulus and task settings, potentially accounting for the previously reported effects. In their study, Pereira and colleagues presented participants with a face, house, and comparison neutral cues, and controlled for stimulus size, distance from the central fixation cross, global luminance, internal configuration of features, attractiveness, background context, and task settings. This is because all of these factors have previously been documented to strongly engage attention, independent of the social nature of faces (size and positioning [46], saliency [47], low-level internal features [48,49,50], valence and attractiveness [51,52,53], and context, [54,55,56]). Pereira and colleagues measured (i) manual responses by examining reaction time to targets that appeared with equal probability at one of the previous cue locations, and (ii) eye movements by examining proportion of saccades towards any of the cue locations. The data revealed no spontaneous attentional biasing towards faces and eyes in manual data and only a small bias in eye movements towards the eyes of the face. Thus, the conclusion from this study was that once stimulus and task factors are tightly controlled, faces and facial features do not spontaneously and robustly bias covert or overt attention.

These findings raise new questions about which stimulus and/or task factors are the most relevant for instantiating a reliable bias of attention towards faces and eyes. In the present study, while continuing to control for both visual content information like global luminance, target-background contrast, and attractiveness, as well as task settings like stimulus distance from the central fixation cross and key-response assignment, we tested whether visual context information in the form of an appropriate background would reinstate social attentional biasing. We reasoned that this manipulation may affect social attention as faces in the real world most often do not appear detached from bodies, isolated from their natural backgrounds, and/or cropped of hair. As such, the lack of social orienting in Pereira and colleagues’ [45] study may have resulted from an artificially high similarity between the comparison face and house cues due to a tight control of these external features across the stimuli. Thus, one possibility is that spontaneous attentional biasing for faces will emerge once a natural background context, likened to how faces are found in the real world, is provided. Past work shows that peripheral situational or background information is important for perceptual and neural processing of faces and objects [57,58,59]. Context is also found to exert strong effects on how social information is prioritized [60,61,62,63], with for example, increased congruency effects in identifying facial emotions when faces are consistent versus inconsistent with background scene contexts [64]. However, it remains relatively unexplored how background context influences social attentional biasing.

**Figure 1:**
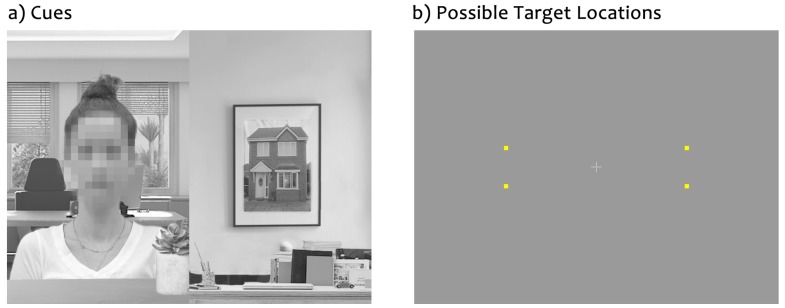
(**a**) The cue screen depicting upright cues with the face in the left visual field. The face has been blurred to preserve the privacy of the actor. (**b**) The target screen depicting all possible target locations for square targets.

To address this question, here we used the same task and parameters as Pereira and colleagues [45], but embedded the face and house cues within natural contextual backgrounds as illustrated in Figure 1. We measured attentional biasing using a dot-probe task and assessed the speed of manual target discrimination when targets were presented at the previous location of the face versus the house cue. Since it is still unclear whether attentional biasing towards faces are driven by faces as a whole or by any specific facial feature, targets were positioned at either the previous location of the eyes or mouth of the face or the top or bottom of the house to allow for a more detailed examination of attentional biasing at each location. Experiment 1 measured covert attention while participants maintained central fixation, whereas Experiment 2 measured natural eye movements using an eye tracker. If contextually-based social information resulted in robust social attentional biasing, we expected to find a reliable social attentional bias in both covert and overt measures, with faster responses in manual measures for targets occurring at the previous location of the face, and in particular the eyes, and greater proportion of saccades directed towards the face and eye cues.

## 2. Experiment 1

### Materials and Methods

**Participants.** Thirty volunteers, with normal or corrected-to-normal vision, participated (25 females, M_age_ = 21 years, SD_age_ = 3 years). They were remunerated with course credits. This sample size falls within the range reflected by an a priori power analysis (G*Power [65]) that was based on the estimated magnitude of face selection effects from past research [24,29,66,67]. The analysis indicated that data from 6–38 participants were needed to detect medium-to-large effects ranging from 0.41–1.36 (as estimated from Cohen’s ƒ) with corresponding power values from 0.95–0.97. Informed consent was obtained from all participants before they participated in the study. The study was conducted in accordance with the Declaration of Helsinki, and all protocol and procedures were approved by the University Research Ethics board (protocol number 81-0909).

**Stimuli and Apparatus.** All stimuli were presented on a 16” cathode ray tube (CRT) monitor at an approximate viewing distance of 60cm. Stimulus presentation sequence was controlled by MATLAB’s psychophysics toolbox [68].

The fixation screen consisted of a fixation cross (1° × 1° of visual angle), positioned at the center of the screen and set against a uniform 60% gray background. The cue stimuli, illustrated in Figure 1, consisted of grey-scale photographs of a female face and a house. The face and house parts of each cue measured 4.2° × 6°, and were positioned 6.3° away from the central fixation cross. A house image was selected as the comparison stimulus due to both faces and houses being canonical stimuli (i.e., those that maintain a consistent internal configuration), with faces containing two eyes and a mouth, and houses typically containing windows and a door. This choice of stimuli maintains consistency with past attentional work [11,69,70,71,72].

Along with size and distance from the fixation cross, the face and house cues were matched for average luminance (computed using the MATLAB SHINE toolbox [73]), Average gray scale luminance (ranging from 0–1) was equated across cues overall (face = 0.60, house = 0.56) as well as between the upper and lower halves of each cue (eyes = 0.60, mouth = 0.60, top house = 0.58, bottom house = 0.55). Michelson contrasts across each of these regions were also equivalent, though some variance existed across the lower half of each cue (eyes = 0.64, mouth = 0.56, top house = 0.65, bottom house = 0.72). Although we did not use a linearized monitor, all luminance and contrast measures reflecting image pixel values were verified to accurately reflect screen measures using a DataColor Spyder3Pro colorimeter.

The face and house cues were also matched for perceived attractiveness (measured via independent raters). Thirty-five additional naïve participants were asked to independently rate images of faces and images of comparison house and object stimuli using a Likert scale ranging from 1—*Very Unattractive* to 10—*Very Attractive*. The cue images used here received equivalent attractiveness ratings, *t*(34) = 1.40, *p* = 0.17, d_z_ = 0.24.

Background context was added to the face and house cues using a photo editing software (Adobe Photoshop CS5), such that the face belonged to a person who was depicted sitting in a room, while the house was depicted as a picture that was hanging on a wall. The target screen consisted of a yellow circle or square (0.3° × 0.3° each), positioned 7.2° away from the fixation cross and set against a uniform 60% gray background.

**Design.** The target discrimination task was a repeated measures design with five factors: *Cue orientation* (upright, inverted), *Face position* (left visual field, right visual field), *Target location* (eyes, mouth, top house, bottom house), *Target identity* (circle, square), and *Cue-target interval* (denoting the time between the onset of the cue and the onset of the target; 250, 360, 560, and 1000 ms).

*Cue orientation* varied between upright and inverted cue images to control for baseline visual differences across the cue stimuli [74,75,76]. *Face position* varied between the left and right visual fields, with the house image always occurring in the opposite visual field. This manipulation was included as previous work has found that social processing of faces is facilitated when they are presented in the left visual field [11,13,14,45,77,78]. *Target location* was varied to occur at either the previous location of the eyes, mouth, top of the house, or bottom of the house. This critical manipulation was included to capture performance differences between targets occurring at the location of the face and its specific facial features relative to the comparison stimuli. *Target identity* was varied between a yellow circle and a yellow square in order to collect both response time (RT) and response accuracy. *Cue-target interval* varied between 250, 360, 560, and 1000 ms in order to assess the time course of attentional biasing and to maintain consistency with past work [24,45].

All factor combinations were equiprobable and presented equally often throughout the task sequence. The cues were spatially uninformative about the target location and its identity, as each target was equally likely to occur at any of the possible target locations. Conditions were intermixed and presented in a randomized order. Thus, participants had no incentive to attend to any particular cue.

**Figure 2:**
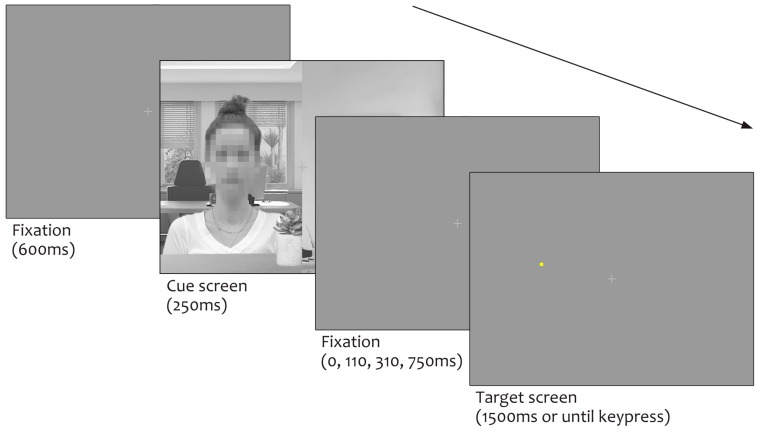
Example trial sequence. Trials began with the presentation of the fixation screen for 600 ms. The cue screen was then presented for 250 ms. After 0, 110, 310, or 750 ms, a target (circle or square) demanding a discrimination response appeared in one of four possible locations. The target remained on screen for 1500 ms or until a key press was made.

**Procedure.** As before [24,45], we used the dot-probe task [79]. Figure 2 depicts the typical sequence of events. After the fixation display of 600 ms, a cue display was shown for 250 ms. After 0, 110, 310, or 750 ms (constituting 250, 360, 560, and 1000 ms cue-target intervals, respectively), a single target was presented at the previous location of the eyes, mouth, top house, or bottom house, and remained visible until participants responded or 1500 ms had elapsed. Participants were instructed to withhold their eye movements and to identify the target by pressing the ‘b’ or ‘h’ keys on the keyboard quickly and accurately (target identity-key response was counterbalanced). They were informed about the progression of the task sequence, that the target was equally likely to be a circle or a square, that the target could appear in any of the possible locations, and that there was no spatial relationship between the cue content, cue orientation, cue placement, target location, or target shape. Participants completed 960 trials divided equally across five testing blocks, with ten practice trials run at the start. Responses were measured from target onset.

## 3. Results

Response anticipations (RTs < 100 ms; 0.3% of all trials), timeouts (RTs > 1000 ms; 2.9%), and incorrect key presses (key press other than ‘b’ or ‘h’; 1.9%) accounted for 5.1% of data and were removed from all analyses. Overall, accuracy was at ceiling at 94% and was not analyzed further.

***Manual RT.*** In order to probe the extent of attentional biasing towards both overall faces and specific facial features (i.e., eyes and mouth), we conducted three sets of analyses. Using null hypothesis significance testing (NHST), we examined mean correct RTs for (1) target responses for the overall face (averaged across target locations of eyes and mouth) compared to the overall house (averaged across target locations of top and bottom house), and (2) target responses for each target location of the eyes, mouth, top house, and bottom house. NHST were performed using repeated measures Analyses of Variance (ANOVA) with Greenhouse-Geiser corrections applied for any violations of sphericity. Paired two-tailed t-tests were used for post-hoc comparisons where applicable, with multiple comparisons corrected using the Holm–Bonferroni procedure to control for Type I error [80]. All comparisons are shown with corresponding adjusted *p*-values (α_FW_ = 0.05 [81]). If background context facilitated social attentional biasing, we expected to find faster responses for targets occurring at the previous location of the face (both overall and/or at the eyes) relative to targets occurring at the previous location of the house.

Furthermore, any null effects were examined using Bayesian analyses to assess (3) the relative strength of evidence for preferential attentional biasing towards faces versus houses by quantifying the evidence for the alternative hypothesis over the null hypothesis [82]. Bayesian analyses were performed using an online Bayes factor calculator (http://www.lifesci.sussex.ac.uk/home/Zoltan_Dienes/inference/bayes_factor.swf) based on previously reported social attentional biasing effects when using similar paradigms. A Bayes factor that is less than 0.33 provides substantial evidence for the null hypothesis, whereas a Bayes factor greater than 3.00 indicates evidence for the alternative hypothesis (values between 0.33 and 3.00 suggest the need for more evidence).

***Overall face* vs. *house comparisons.*** Mean correct interparticipant RTs were analyzed using an omnibus repeated measures ANOVA, run as a function of *Cue orientation* (upright, inverted), *Face position* (left visual field, right visual field), *Target location* (face, house), and *Cue-target interval* (250, 360, 560, and 1000 ms). There was a main effect of *Cue-target interval* [*F*(3,87) = 62.31, *p* < 0.001, η_p_^2^ = 0.68], indicating overall faster RTs for longer relative to shorter cue-target intervals [250 ms vs. all, *t >* 9.80, *p*s < 0.001, *d_z_*s > 1.79; 360 ms vs. all, *t*s > 3.20, *p*s < 0.008, *d_z_*s > 0.58; all other *p* = 0.36, *d_z_* = 0.17]. This finding demonstrates the typical foreperiod effect [83,84], reflecting increased preparation to respond with a lengthening of the time between the cue and target. As such, this finding shows that participants performed the task with an expected degree of preparation and alertness to the target. Importantly though, no effects of *Target location* were found [*F*(1,29) = 3.73, *p* = 0.06, η_p_^2^ = 0.11].

Two interactions with Target location reached significance. A two-way interaction between *Target location* and *Cue-target interval* [*F*(3,87) = 3.25, *p* = 0.026, η_p_^2^ = 0.10] indicated slower RTs for targets that occurred at the previous location of the face vs. house cue at a cue-target interval of 560 ms [*t*(29) = 3.11, *p* = 0.017, *d_z_* = 0.57; all other *p*s > 0.13, *d_z_*s < 0.39]. A three-way interaction between *Face position*, *Target location*, and *Cue-target interval* [*F*(3,87) = 4.96, *p* = 0.003, η_p_^2^ = 0.15] was reliable as well. When separated by Face position, significant main effects of *Cue-target interval* were found when the face was presented in both the left and right visual fields [*F*(3,87) = 36.85, *p* < 0.001, η _p_^2^ = 0.56; *F*(3,87) = 57.87, *p* < 0.001, η_p_^2^ = 0.67, respectively], showing faster RTs for longer relative to shorter cue-target intervals [left visual field, 250 ms vs. all, *t*s > 6.90, *p*s < 0.001, *d_z_*s > 1.26; 360 ms vs. 1000 ms, *t*(29) = 3.05, *p* = 0.014, *d_z_* = 0.56; all other *p*s > 0.07, *d_z_*s < 0.40; right visual field, 250 ms and 360 ms vs. all, *t*s > 2.43, *p*s < 0.043, *d_z_*s > 0.44; all other *p* = 0.94, *d_z_* = 0.01]. When faces were presented in the left visual field, an interaction between *Target location* and *Cue-target interval* [*F*(3,87) = 8.18, *p* < 0.001, η_p_^2^ = 0.22] further indicated slower RTs for targets occurring at the previous location of the face vs. house cue at 560 ms [*t*(29) = 3.13, *p* = 0.016, *d_z_* = 0.57; all other *p*s > 0.12, *d_z_*s < 0.39]. No other significant main effects or interactions were found [*F*s < 3.94, *p*s > 0.06, η_p_^2^ < 0.12].

**Figure 3:**
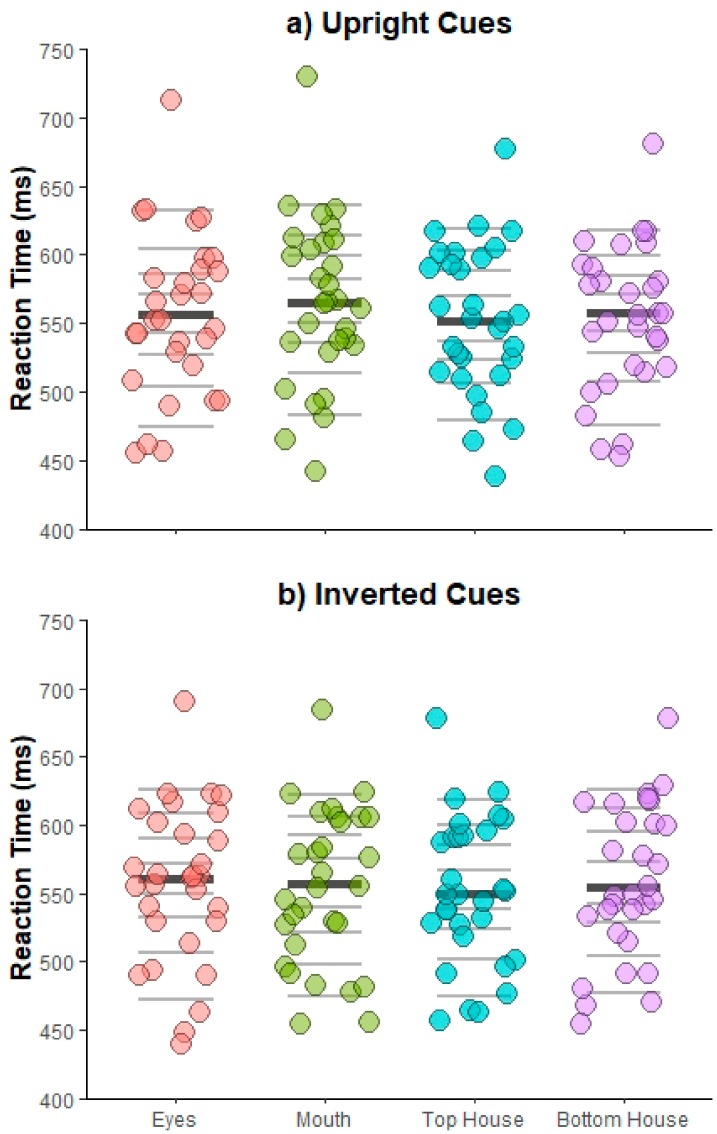
Experiment 1 results. Stripcharts depicting mean correct response times (RTs) for each participant as a function of Target position for Upright (**a**) and Inverted (**b**) cues. Horizontal lines mark the deciles, with the thicker darker line representing the median. Note that the reported pattern of results does not vary even if the outlier is removed from analyses.

***Specific facial features* vs. *house comparisons.*** Mean correct interparticipant RTs were analyzed using an omnibus repeated measures ANOVA, run as a function of *Cue orientation* (upright, inverted), *Face position* (left visual field, right visual field), *Target location* (eyes, mouth, top house, bottom house), and *Cue-target interval* (250, 360, 560, 1000 ms). Figure 3 illustrates mean RTs for each participant as a function of target position for Upright (3a) and Inverted (3b) cues.

The results revealed main effects of *Cue-target interval* [*F*(3,87) = 62.09, *p* < 0.001, η_p_^2^ = 0.68] and *Target location* [*F*(3,87) = 2.96, *p* = 0.037, η_p_^2^ = 0.09]. The first indicated overall faster RTs for longer relative to shorter cue-target intervals [250 ms vs. all, *t*s > 9.70, *p*s < 0.001, *d_z_*s > 1.77; 360 ms vs. all, *t*s > 3.17, *p*s < 0.007, *d_z_*s > 0.58; all other *p* = 0.36, *d_z_* = 0.17], demonstrating the typical foreperiod effect [83,84]. The second main effect indicated overall slower RTs for targets that occurred at the previous location of the mouth vs. top house cues [*t*(29) = 3.01, *p* = 0.032, *d_z_* = 0.55; all other *ts* < 1.67, *ps* > 0.53, *d_z_*s < 0.31], with no facilitative effects for the eyes in comparison to the house cues [*ts* < 1.39, *ps* > 0.53, *d_z_*s < 0.25]. A two-way interaction between *Cue orientation* and *Target location* [*F*(3,87) = 3.20, *p* = 0.027, η_p_^2^ = 0.10] further showed that this finding held only for upright cues [*t*(29) = 3.61, *p* = 0.007, *d_z_* = 0.66; all other *p*s > 0.05, *d_z_*s < 0.50; inverted cues, all *p*s > 0.19, *d_z_*s < 0.41]. 

A three-way interaction between *Face position*, *Target location*, and *Cue-target interval* was reliable as well [Mauchly’s test of sphericity, χ^2^(44) = 63.56, *p* = 0.03; *F*(6.41,185.89) = 2.33, *p* = 0.031, η_p_^2^ = 0.07]. When run separately by Face position, significant main effects of *Cue-target interval* for both the left and right visual field were found [*F*(3,87) = 37.32, *p* < 0.001, η_p_^2^ = 0.56; *F*(3,87) = 57.52, *p* < 0.001, η_p_^2^ = 0.67, respectively], with faster RTs for longer relative to shorter cue-target intervals [left visual field, 250 ms vs. all, *t*s > 6.98, *p*s < 0.001, *d_z_*s > 1.27; 360 ms vs. 1000 ms, *t*(29) = 3.04, *p* = 0.015, *d_z_* = 0.55; all other *p*s > 0.07, *d_z_*s < 0.40; right visual field, 250 ms and 360 ms vs. all, *t*s > 2.42, *p*s < 0.044, *d_z_*s > 0.44; all other *p* = 0.92, *d_z_* = 0.02]. Furthermore, a significant interaction between *Target location* and *Cue-target interval* [Mauchly’s test of sphericity, χ^2^(44) = 62.23, *p* = 0.04; *F*(6.15,178.25) = 3.08, *p* = 0.006, η_p_^2^ = 0.10] was found when faces were presented in the left visual field, indicating slower RTs for targets occurring at the previous location of the eyes vs. top house cue at 560 ms only [*t*(29) = 3.22, *p* = 0.019, *d_z_* = 0.59; all other *p*s > 0.13, *d_z_*s< 0.43]. No other effects were reliable [*F*s < 2.09, *p*s > 0.10, η_p_^2^ < 0.07].

***Bayesian analyses.*** To further examine the plausibility of no attentional differences between the cues, we performed Bayesian analyses using a two-tailed Gaussian distribution centered around a mean of 17.67 ms and SD of 7.55 ms, which reflected the previously-reported manual RT advantage for faces vs. objects ([24]; Experiments 1a,b). A Bayes factor of 0.08 was found for upright face vs. house contrasts, thus supporting the findings from the NHST and providing evidence in favor of the null hypothesis of no difference in reaction times between the face and house cues.

## 4. Discussion

If contextually-based social information resulted in spontaneous covert social attentional biasing, we expected to find faster responses for targets occurring at the previous location of the face overall and/or the eyes specifically. Our data did not support this hypothesis, indicating no attentional effects for targets occurring at the location of the face or the eyes. If anything, there was a short-lived effect at 560 ms cue-target interval only, suggesting slower RTs for overall faces relative to houses, as well as specifically for eyes relative to top house, when faces were presented in the left visual field; however, since this finding was not specific to upright faces, it may have reflected differences in the stimulus properties of the contextualized cues [74,75,76]. Similar contextualized differences may have been responsible for slower RTs for the mouth relative to top house targets, both overall and when cues were presented in an upright orientation, particularly since this effect was not specific to when faces were presented in the left visual field. Additionally, Bayes analyses supported the null hypothesis of no differences between face and house cues.

Experiment 1 then suggests that when the face and house stimuli are presented within appropriate background context, there are no reliable effects to indicate preferential covert attentional biasing towards the face or the eyes. These results are consistent with our recent work [45], and further suggest that covert social attention is not determined by contextual factors alone. In Experiment 2, we examined whether these results held when we measured overt attention.

## 5. Experiment 2

In the Pereira and colleagues [45] study, when participants were allowed to make eye movements during the dot-probe task, they broke central fixation on 11% of all trials. Of these 11% of trials, when examining where saccades were directed, it was found that participants looked towards the eyes of the face 17% of the time. This reliable, albeit modest, bias to look at the eyes reflects a potential dissociation between covert and overt orienting towards social stimuli. In the present experiment, we examined whether similar oculomotor biasing also occurred when cues were presented within contextual backgrounds. To do so, we did not provide participants with any instructions to maintain central fixation, but measured their spontaneous eye movements while they performed the same dot probe task as in Experiment 1. 

### Materials and Methods

**Participants, Apparatus, Stimuli, Design, and Procedure.** Thirty new volunteers (23 females, M_age_ = 21 years, SD_age_ = 3 years) participated. None took part in the previous experiment and all reported normal or corrected-to-normal vision. All stimuli, design, and procedures were identical to Experiment 1, except that: (i) participants’ eye movements were tracked using a remote EyeLink 1000 eye tracker (SR Research; Mississauga, ON) recording with a sampling rate of 500 Hz and a spatial resolution of 0.05°. Although viewing was binocular, only the right eye was tracked; (ii) prior to the start of the experiment, a nine-point calibration was performed, and spatial error was rechecked before every trial using a single-point calibration dot. Average spatial error was no greater than 0.5°, with maximum error not exceeding 1°; and (iii) participants were not given any instructions regarding maintaining central fixation in order to preserve their natural eye movements during the task.

## 6. Results

Anticipations (0.1%), timeouts (2.2%), and incorrect key presses (0.1%) were removed from manual data analyses. Overall response accuracy was 96%. Manual RTs were analyzed as before using the same three sets of analyses.

***Overall face* vs. *house comparisons.*** Mean correct RTs were analyzed using an omnibus ANOVA, run as a function of *Cue orientation* (upright, inverted), *Face position* (left visual field, right visual field), *Target location* (face, house), and *Cue-target interval* (250, 360, 560, and 1000 ms). A significant main effect of *Cue-target interval* [Mauchly’s test of sphericity, χ^2^(5) = 14.72, *p* = 0.012; *F*(2.22,64.50) = 62.95, *p* < 0.001, η_p_^2^ = 0.69] emerged, with overall slower RTs for short vs. longer cue-target intervals [250 ms vs. all, *t*s > 8.18, *p*s < 0.001, *d_z_*s > 1.49; all other *p*s > 0.34, *d_z_*s < 0.30]. However, similar to the overall comparisons for Experiment 1, no effects of *Target location* were found [*F*(1,29) = 0.81, *p* = 0.38, η_p_^2^ = 0.03]. 

A significant two-way interaction between *Cue orientation* and *Target location* [*F*(1,29) = 6.73, *p* = 0.015, η_p_^2^ = 0.19] indicated a numerical pattern of slower RTs for inverted vs. upright houses, though post-hoc comparisons did not reach significance [all *p*s > 0.06, *d_z_*s < 0.42]. Additionally, a three-way interaction between *Cue orientation*, *Target location*, and *Cue-target interval* [*F*(3,87) = 3.08, *p* = 0.032, η_p_^2^ = 0.10] emerged once again. When separated by *Cue orientation*, there was a significant main effect of *Cue-target interval* for both upright and inverted cues [Mauchly’s test of sphericity, χ^2^(5) = 12.79, *p* = 0.026; *F*(2.37,68.82) = 40.60, *p* < 0.001, η_p_^2^ = 0.58; and *F*(3,87) = 47.54, *p* < 0.001, η_p_^2^ = 0.62, respectively], with overall slower RTs for short vs. longer cue-target intervals [upright, 250 ms vs. all, *t*s > 6.90, *p*s < 0.001, *d_z_*s > 1.26; all other *p*s > 0.62, *d_z_*s < 0.24; inverted, 250 ms vs. all, *t*s > 7.53, *p*s < 0.001, *d_z_*s > 1.37; all other *p*s > 0.23, *d_z_*s < 0.34]. Furthermore, a significant main effect for *Target location* [*F*(1,29) = 5.17, *p* = 0.031, η_p_^2^ = 0.15] was found for inverted cues, with slower RTs for overall faces vs. houses. An interaction between *Target location* and *Cue-target interval* [*F*(3,87) = 2.92, *p* = 0.039, η_p_^2^ = 0.09] was found for upright cues, indicating a numerical pattern of faster RTs for faces vs. houses at 250 ms only, though post-hoc comparisons did not reach significance [all *p*s > 0.06, *d_z_*s < 0.47]. No other significant main effects or interactions were found [*F*s < 1.64, *p*s > 0.19, η_p_^2^ < 0.05].

**Figure 4:**
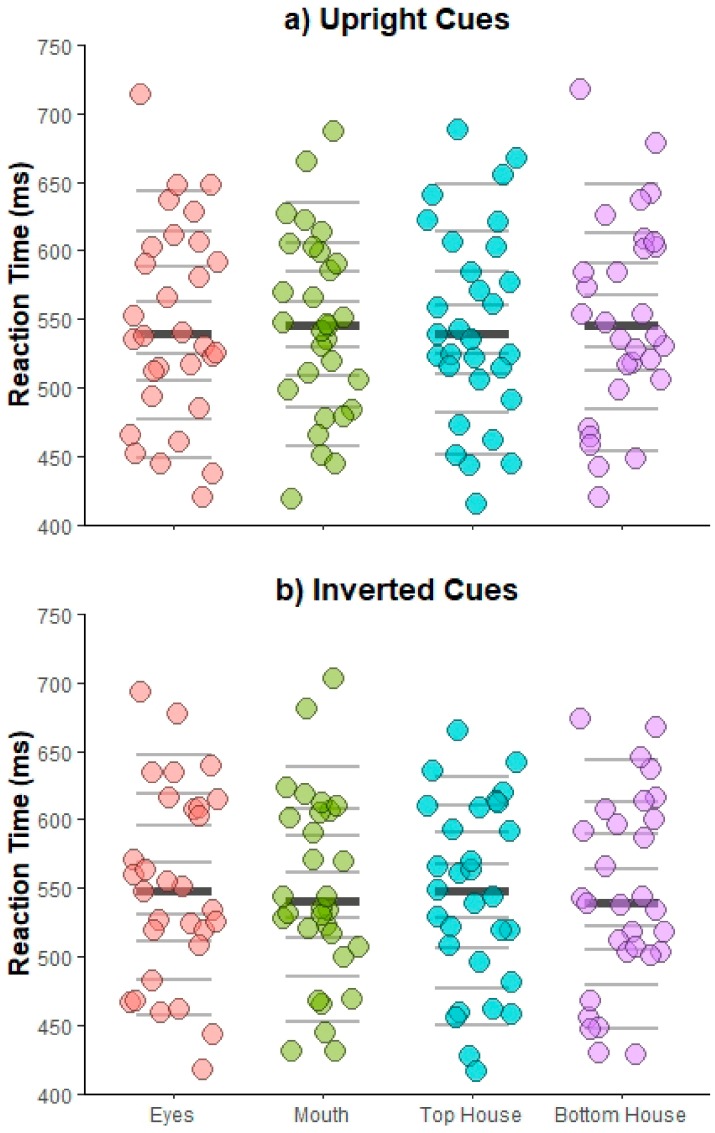
Experiment 2 manual results. Stripcharts depicting mean correct RTs for each participant as a function of target position for Upright (**a**) and Inverted (**b**) cues. Horizontal lines mark the deciles, with the thicker darker line representing the median.

***Specific facial features* vs. *house comparisons.*** An omnibus ANOVA with *Cue orientation* (upright, inverted), *Face position* (left visual field, right visual field), *Target position* (eyes, mouth, top house, and bottom house), and *Cue-target interval* (250, 360, 560, 1000 ms) was run. Mean RTs for each participant are illustrated in Figure 4 for Upright (4a) and Inverted (4b) cues.

Similar to the pattern of results found for overall faces vs. houses, the ANOVA indicated a main effect of *Cue-target interval* [Mauchly’s test of sphericity, χ^2^(5) = 14.04, *p* = 0.015; *F*(2.24,65.07) = 62.30, *p* < 0.001, η_p_^2^ = 0.68], which was once again driven by overall slower RTs at shorter cue-target times [250 ms vs. all, *t*s > 8.10, *p*s < 0.001, *d_z_*s > 1.48; all other *p*s > 0.35, *d_z_*s < 0.30] and a significant interaction between *Cue orientation* and *Target location* [*F*(3,87) = 3.02, *p* = 0.034, η_p_^2^ = 0.09], indicating a numerical pattern of shorter RTs for eyes for upright vs. inverted faces and shorter RTs for bottom house for inverted vs. upright houses, though post-hoc comparisons did not reach significance [all *p*s > 0.07, *d_z_*s < 0.49]. No other effects were found [*F*s < 1.89, *p*s > 0.06, η_p_^2^ < 0.06].

***Bayesian analyses.*** Once again, Bayes factor was used to examine the plausibility of these findings using the same parameters as before (i.e., two-tailed Gaussian distribution, M = 17.67, SD = 7.55; ([24]; Experiments 1a,b)). A Bayes factor of 0.07 was found for upright face vs. house contrasts, which once again provided support for the null over the alternative hypothesis indicating no difference in reaction times between the face and house cues.

**Figure 5:**
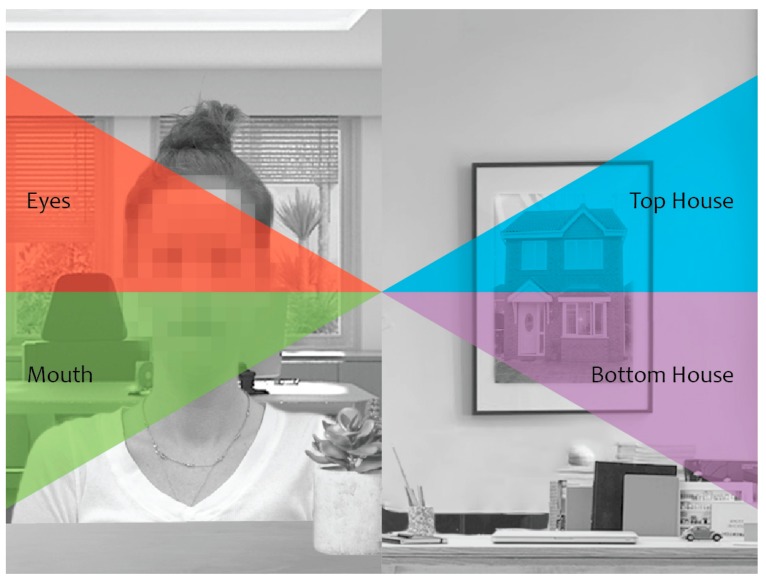
Regions of interest (ROI). ROIs were defined by a radial window that included the area of interest; red = eyes, green = mouth, blue = top house, and purple = bottom house.

**Oculomotor data.** To assess if participants spontaneously looked at the face cue more frequently, we next examined trials in which saccades were launched from central fixation towards one of the predefined regions of interest (ROI), i.e., eyes, mouth, top house, or bottom house location, during the 250 ms cue period only, as we were specifically interested in examining attentional biasing in response to the cue stimuli. As illustrated in Figure 5, each ROI was comprised of its respective cue region and spanned a 30° radial window. Saccades were defined as eye movements with an amplitude of at least 0.5°, an acceleration threshold of 9500°/s^2^, and a velocity threshold of 30°/s.

For each participant, we calculated the proportion of saccades for each ROI by examining the direction of the very first saccade that was launched from central fixation towards one of the ROIs upon cue onset. The number of saccades that were launched towards each ROI were tallied across the entire experiment for each participant and then divided by the total number of first saccades that occurred during the cue period. On average, participants saccaded away from the fixation cross on 11% of all trials, of which saccades were launched towards an ROI on 91% of those trials. As with manual RT, we conducted NHST to analyze the proportion of saccades launched towards (1) the overall face versus the house and (2) each specific target location (eyes, mouth, top house, bottom house), and we conducted Bayesian analyses to examine any null effects to assess (3) the relative strength of evidence for the alternative over the null hypothesis.

***Overall face* vs. *house comparisons.*** Proportion of saccades were analyzed using a repeated measures ANOVA run as a function of *Cue orientation* (upright, inverted), *Face position* (left visual field, right visual field), and *ROI* (face, house). Main effects of *Cue orientation* [*F*(1,29) = 13.23, *p* = 0.001, η_p_^2^ = 0.31] and *Face position* [*F*(1,29) = 9.90, *p* = 0.004, η_p_^2^ = 0.25] were reliable, with a greater proportion of saccades occurring when cues were upright and when faces were presented in the left visual field, respectively.

However importantly, there was a significant main effect of *ROI* [*F*(1,29) = 51.96, *p <* 0.001, η_p_^2^ = 0.64], with an overall greater proportion of saccades towards the face compared to the house. This main effect was further qualified by a significant interaction between *Cue orientation* and *ROI* [*F*(1,29) = 15.84, *p* < 0.001, η_p_^2^ = 0.35], which demonstrated a larger bias for proportion of saccades towards the face vs. house for upright cues [*t*(29) = 6.53, *p* < 0.001, *d_z_* = 1.19] as compared to inverted cues [*t*(29) = 3.68, *p* = 0.001, *d_z_* = 0.67]. An interaction between *Face position* and *ROI* [*F*(1,29) = 6.85, *p* = 0.014, η_p_^2^ = 0.19] further demonstrated a larger effect for the proportion of saccades towards the face vs. house when the face was presented in the left visual field [*t*(29) = 5.80, *p* < 0.001, *d_z_* = 1.06] as compared to the right visual field [*t*(29) = 3.01, *p* = 0.005, *d_z_* = 0.55]. No other significant effects were found [*F*s < 3.43, *p*s > 0.07, η_p_^2^s < 0.11].

**Figure 6:**
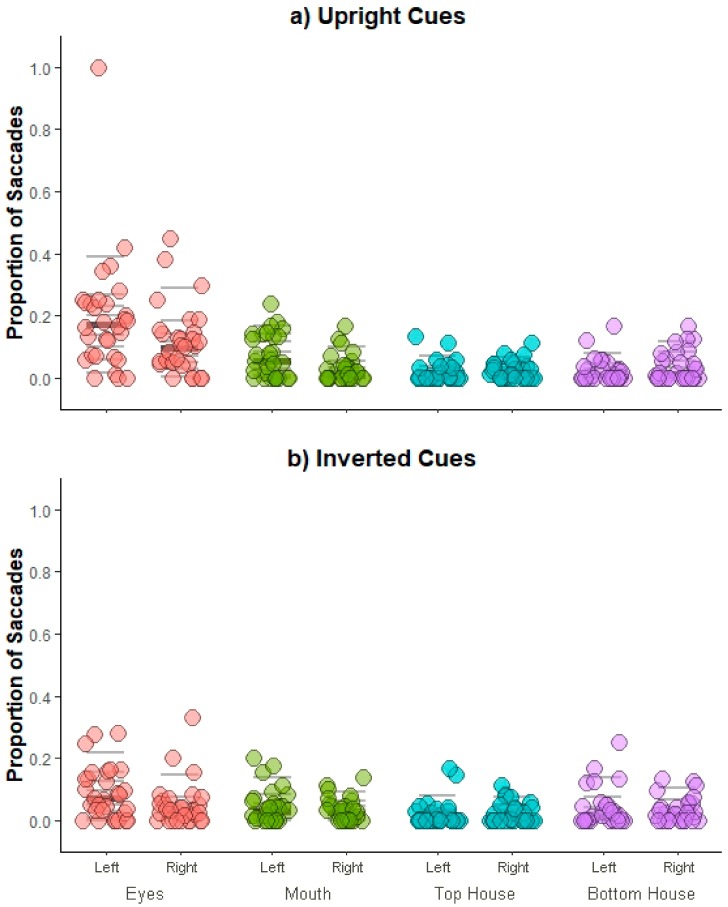
Experiment 2 eye movement results. Stripcharts depicting mean proportion of saccades for each participant during the cue presentation period as a function of face position and ROI for Upright (**a**) and Inverted (**b**) cues. Horizontal lines mark the deciles, with the thicker darker line representing the median. Note that the pattern of results does not change even if the outlier is removed from analyses.

***Specific facial features* vs. *house comparisons.*** Proportion of saccades were examined using a repeated measures ANOVA run as a function of *Cue orientation* (upright, inverted), *Face position* (left visual field, right visual field), and *ROI* (eyes, mouth, top house, bottom house). Mean proportion of saccades away from the fixation cross are illustrated in Figure 6 as a function of ROIs for Upright (6a) and Inverted (6b) cues. 

Similar to the overall comparisons, there were main effects of *Cue orientation* [*F*(1,29) = 13.23, *p* = 0.001, η_p_^2^ = 0.31] and *Face position* [*F*(1,29) = 9.90, *p* = 0.004, η_p_^2^ = 0.25], showing that a greater proportion of saccades occurred when cues were upright and when faces were presented in the left visual field, respectively. Importantly, we also found a main effect of *ROI* [Mauchly’s test of sphericity, χ^2^(5) = 25.89, *p* < 0.001; *F*(1.92,55.54) = 43.53, *p <* 0.001, η_p_^2^ = 0.60], with an overall greater proportion of saccades towards the eyes compared to all other ROIs [*t*s > 6.79, *p*s < 0.001, *d_z_*s > 1.24] and an overall great proportion of saccades towards the mouth compared to top house [*t*(29) = 4.06, *p* = 0.001, *d_z_*= 0.74; all other *p*s > 0.07, *d_z_*s < 0.41].

This main effect was further qualified by a significant interaction between *Cue orientation* and *ROI* [Mauchly’s test of sphericity, χ^2^(5) = 35.91, *p* < 0.001; *F*(1.67,48.54) = 8.49, *p* = 0.001, η_p_^2^ = 0.23]. When cues were upright, a greater proportion of saccades were directed towards the Eyes compared to all other regions [*t*s > 4.72, *p*s < 0.001, *d_z_*s > 0.87], along with greater proportion of saccades towards the mouth compared to top house [*t*(29) = 3.03, *p* = 0.015, *d_z_* = 0.55; all other *p*s > 0.17, *d_z_*s < 0.33]. A similar pattern was found when cues were inverted, however this effect was numerically smaller and was specific to the eye region only [eyes vs. all other regions, *t*s > 2.72, *p*s < 0.04, *d_z_*s > 0.50; all other *p*s > 0.16, *d_z_*s < 0.37]. A reliable *Face position* and *ROI* interaction emerged as well [Mauchly’s test of sphericity, χ^2^(5) = 59.00, *p* < 0.001; *F*(1.44,41.81) = 4.52, *p* = 0.027, η_p_^2^ = 0.14], which further suggested that proportion of saccades towards the eyes and mouth was greater when faces were presented in the left visual field. That is, a greater proportion of saccades were launched towards the eyes compared to all other regions and the mouth compared to top house when the face was presented in the left visual field [*t*s > 3.71, *p*s < 0.003, *d_z_*s > 0.68; all other *p*s > 0.05, *d_z_*s < 0.43]; however, this effect was smaller and only specific to the eyes when the face was presented in the right visual field [eyes vs. all other regions, *t*s > 3.32, *p*s < 0.01, *d_z_*s < 0.61; all other *p*s > 0.14, *d_z_*s < 0.38]. No other effects were found [*F* < 1.12, *p*s > 0.30, η_p_^2^ < 0.04].

Thus, when participants’ natural eye movements were measured, spontaneous saccades were launched more frequently towards the face overall as well as the eyes specifically, particularly when the face was presented in an upright orientation and when it was positioned in the left visual field. 

## 7. Discussion

In Experiment 2, we examined whether participants’ overt attention was spontaneously directed toward faces or their specific features. Without any specific instructions about eye movements, we once again found no manual advantages for targets occurring at the location of the face and Bayesian analyses provided evidence for the null hypothesis of no RT differences between targets occurring at the previous location of the face and house cues. However, when we examined spontaneous eye movements, we found that participants broke fixation and looked at the cue stimuli on 11% of all trials, which is numerically consistent with the percentage of saccades found in the Pereira and colleagues [45] study. However here, saccades were launched towards the eye region on 48% (versus 17% in the previous study) of trials that broke fixation. This finding was also qualified by an increase in the proportion of saccades towards faces overall, and eyes specifically, when faces were upright and when they were presented in the left visual field. Therefore, even though oculomotor biasing occurred on a small subset of all trials, it appears that faces presented within consistent contextual backgrounds exert differential effects across manual and overt responses.

## 8. General Discussion

The present study examined whether social information presented in context influenced spontaneous social attention biasing. Using the dot-probe paradigm, we presented participants with face and house cues embedded within appropriate contextual backgrounds and measured their speed of target discrimination when targets were presented at the previous location of the face (eyes, mouth) versus the house (top, bottom). While controlling for stimulus information across size, distance from the fixation cross, overall luminance, and attractiveness between the face and house stimuli (as in Pereira and colleagues’ [45] study), we measured covert attention by instructing participants to maintain central fixation in Experiment 1 and spontaneous eye movements by using an eye tracker in Experiment 2.

No evidence of attentional biasing towards faces or facial features was found in manual responses in either experiment. This replicates and extends our previous work demonstrating that covert social attentional biasing is fragile in nature and affected by stimulus content factors [45] even when the stimuli are embedded in appropriate background contexts. Thus, visual context alone appears to be insufficient in engaging social attention biasing in covert measures. However, when we measured participants’ eye movements, we found that their overt attention was biased towards the eyes of faces when they were presented in an upright orientation and in the left visual field. Although this biasing towards the eye region occurred in only 48% of trials in which participants broke fixation during the cue display (i.e., only 5.3% of all trials), the magnitude of this effect was numerically larger than in Pereira and colleagues’ [45] study, where they observed biasing towards the eye region on only 17% of trials in which participants broke fixation (i.e., 1.9% of all trials). This suggests that it may be quicker and less effortful to extract social information from faces when they are presented in the appropriate context. However, since these observations are based on between-study comparisons, future investigations are needed in which background context is directly manipulated using a within-participants design to arrive at a more precise estimation of the effects of context on the magnitude of social attention biasing. Taken together, the results of the present study show that contextually-embedded social information does not result in spontaneous social attentional biasing in covert measures but does appear to modulate the magnitude of attentional biasing in overt measures.

These findings raise three main discussion points. One, they suggest that past work that has reported robust effects of social attention biasing in manual and oculomotor measures when using more uncontrolled stimuli [24,25,29,30,32,36,37,67] likely did not reflect the contribution of visual context alone. Instead, it is more plausible that these effects were due to some combination of visual context, stimulus content, and task factors. Content factors such as luminance, internal configuration of features, and emotional valence have each been documented to engage attention irrespective of any biases elicited by the social nature of faces [47,48,50,85,86]. Additional factors, like geometrical shape, that are specific to faces but not tied to any inherent social importance that faces contain may also play a role in attentional biasing towards these social stimuli [87]. Furthermore, task settings, like the predictability of the cues and the setting of the attentional paradigms have also been found to modulate the magnitude of social attentional effects [83,88]. For example, Burra, Framorando, and Pegna [89] investigated the electrophysiological correlates of eye gaze processing and found that perceiving eye gaze was highly dependent on whether the faces were relevant to the task. Similarly, Hessels and colleagues [90] engaged participants in face-to-face communication and found that gaze allocation was affected by task instructions (i.e., speaking versus listening) and the social context of the communication (i.e., direct conversation versus pre-recorded video). Dovetailing with these data, the present results point to the underlying influence of both stimulus and task settings in spontaneous attentional biasing towards faces and eyes, and highlight the need for future investigations geared towards manipulating and isolating the contribution of visual context, stimulus content, and task factors.

Two, while overt measures demonstrated infrequent effects, they were nevertheless statistically reliable. This is consistent with recent work by Hayward and colleagues [43] who compared social biasing occurring within a typical cuing task with social biasing occurring during a live social interaction. One difference that emerged in the comparison of these methods was the relative scarcity of gaze following observed during real-world interaction. Subsequently, Blair, Capozzi, and Ristic [91] found similarly infrequent though reliable effects when examining overall social orienting during gaze cuing tasks. Together, these data demonstrate that gaze following and social orienting may in actuality occur relatively infrequently, which further suggests that these behaviors may be contextually and situationally mediated, such that appropriate attentional responses only need to occur occasionally in order to affect behavior reliably. Our eye movement measures support these findings showing that orienting may be reflective of an infrequent bias towards key parts of social cues. 

Finally, while social attention biasing was observed in overt measures, no effects emerged in covert measures. This result adds to the growing body of evidence demonstrating dissociations between covert and overt measures of social attention, in that the two modes of orienting appear to serve different purposes in real-world social environments—covert attention is hypothesized to serve as a mechanism that surreptitiously gathers information from the environment, while overt attention is hypothesized to serve as an active signaling mechanism in order to communicate with others [44,92,93,94,95]. These dissociations have only just begun to be probed on an experimental level [42,96,97,98,99], with the present study along with Pereira and colleagues’ [45] study providing direct evidence in support of this distinction. Future studies in which covert and overt attention are systematically manipulated and measured are needed to understand the nature of this dissociation.

In sum, the present investigation shows that spontaneous social attention biasing may diverge across covert and overt measures. This underscores the fragility of spontaneous attentional biasing towards social information and points to the need for systematic investigations of the specific contributions of stimulus content and visual context factors in covert and overt social attention.
